# Rectus femoris graft harvest for anterior cruciate ligament reconstruction does not result in selective rectus femoris muscle volume loss or strength deficit at one year: quantitative magnetic resonance imaging, isokinetic and functional analyses

**DOI:** 10.1007/s00264-026-06886-0

**Published:** 2026-06-06

**Authors:** Ahmet Emin Okutan, Lokman Kehribar, Enes Gürün, Ali Kerim Yılmaz

**Affiliations:** 1 Dept. of Orthopaedics, Samsun University, Samsun, Turkey; 2 Dept. of Orthopaedics, Dokuz Eylül University, Izmir, Turkey; 3 Dept. of Radiology, Samsun University, Samsun, Turkey; 4 Dept. of Sports Science, Ondokuz Mayıs University, Samsun, Turkey

**Keywords:** Anterior cruciate ligament reconstruction, Rectus femoris tendon, Autograft, Quadriceps atrophy, Isokinetic strength

## Abstract

**Purpose:**

To evaluate 1-year clinical and functional outcomes, as well as donor-site morbidity based on quadriceps muscle morphology, after primary single-bundle ACLR using a quadrupled RF tendon autograft.

**Methods:**

Patients who underwent primary single-bundle ACLR using a quadrupled RF tendon autograft with adjustable suspensory fixation between December 2024 and April 2025 were retrospectively reviewed. Clinical outcomes were assessed using the International Knee Documentation Committee Subjective Knee Form (IKDC), Marx Activity Scale, KT-1000 arthrometer side-to-side difference, and pivot-shift test. Quadriceps muscle volumes, including RF, vastus medialis (VM), vastus lateralis (VL), and vastus intermedius (VI), were measured using magnetic resonance imaging (MRI) at one year postoperatively. Isokinetic strength testing at 60°/s and 240°/s and hop performance tests were performed to determine limb symmetry indexes (LSI).

**Results:**

A total of 54 patients were evaluated with a mean follow-up of 15.7 ± 4.2 months. The mean IKDC score was 84.1 ± 9.7, the mean Marx Activity Scale score was 7.1 ± 3.2, and the mean side-to-side anterior tibial translation difference was 1.8 ± 1.2 mm. All quadriceps muscle volumes were significantly lower in the operated limb than in the contralateral limb (*p* < 0.001). Mean deficits were 9.63% for RF, 8.52% for VL, 7.95% for VI, and 8.08% for VM. No significant difference was observed among the percentage deficits of the four muscles (F = 1.701, *p* = 0.170). LSI values exceeded 90% in all evaluated strength and functional parameters. No significant side-to-side differences were found in most isokinetic and hop tests, except for 240°/s extension strength (*p* = 0.03) and triple-hop performance (p < 0.001).

**Conclusion:**

Primary ACLR using a quadrupled RF tendon autograft resulted in favourable one year clinical and functional outcomes with restoration of limb symmetry. Quantitative MRI analyses demonstrated that RF graft harvest did not result in selective RF muscle volume loss or clinically relevant strength deficit at one year postoperatively.

## Introduction

Anterior cruciate ligament (ACL) reconstruction (ACLR) remains one of the most commonly performed procedures in orthopaedic sports medicine, with autografts representing the gold standard due to their superior biological integration and long-term reliability [1, 2]. Among available options, hamstring, quadriceps and patellar tendon grafts are most frequently used, each offering distinct biomechanical and surgical advantages [3–5]. However, contemporary ACLR is increasingly shifting toward patient-specific graft selection, emphasizing not only graft strength and stability but also donor-site preservation and functional recovery [5]. In this context, the rectus femoris (RF) tendon has emerged as a promising alternative autograft [6]. Its favourable biomechanical properties, together with adequate length and diameter, enable versatile use in both primary and revision ACL reconstruction, allowing flexible graft configurations and combined procedures with a single harvest, while potentially minimizing disruption of the extensor mechanism [7–9].

Despite these advantages, donor-site morbidity remains a key concern in graft selection, particularly when harvesting from the quadriceps mechanism [10, 11]. Early evidence has demonstrated comparable quadriceps performance at six months following ACL reconstruction with RF and hamstring tendon autografts, suggesting that RF tendon harvest may not impair early strength recovery [12]. However, the overall donor-site impact of this technique remains insufficiently characterized. In particular, there is a lack of comprehensive data evaluating structural muscle changes and their relationship to functional outcomes, representing an important gap in the current literature.

Therefore, the aim of the present study was to investigate donor-site morbidity following RF graft harvest for ACL reconstruction using a comprehensive, multimodal approach. Muscle volume was quantitatively assessed using magnetic resonance imaging (MRI), while strength and functional outcomes were evaluated through isokinetic and functional analyses, with side-to-side comparison to the contralateral limb. It was hypothesized that RF graft harvest would not result in selective RF muscle volume loss or strength deficit at one year follow-up.

## Material and methods

**Fig. 1:**
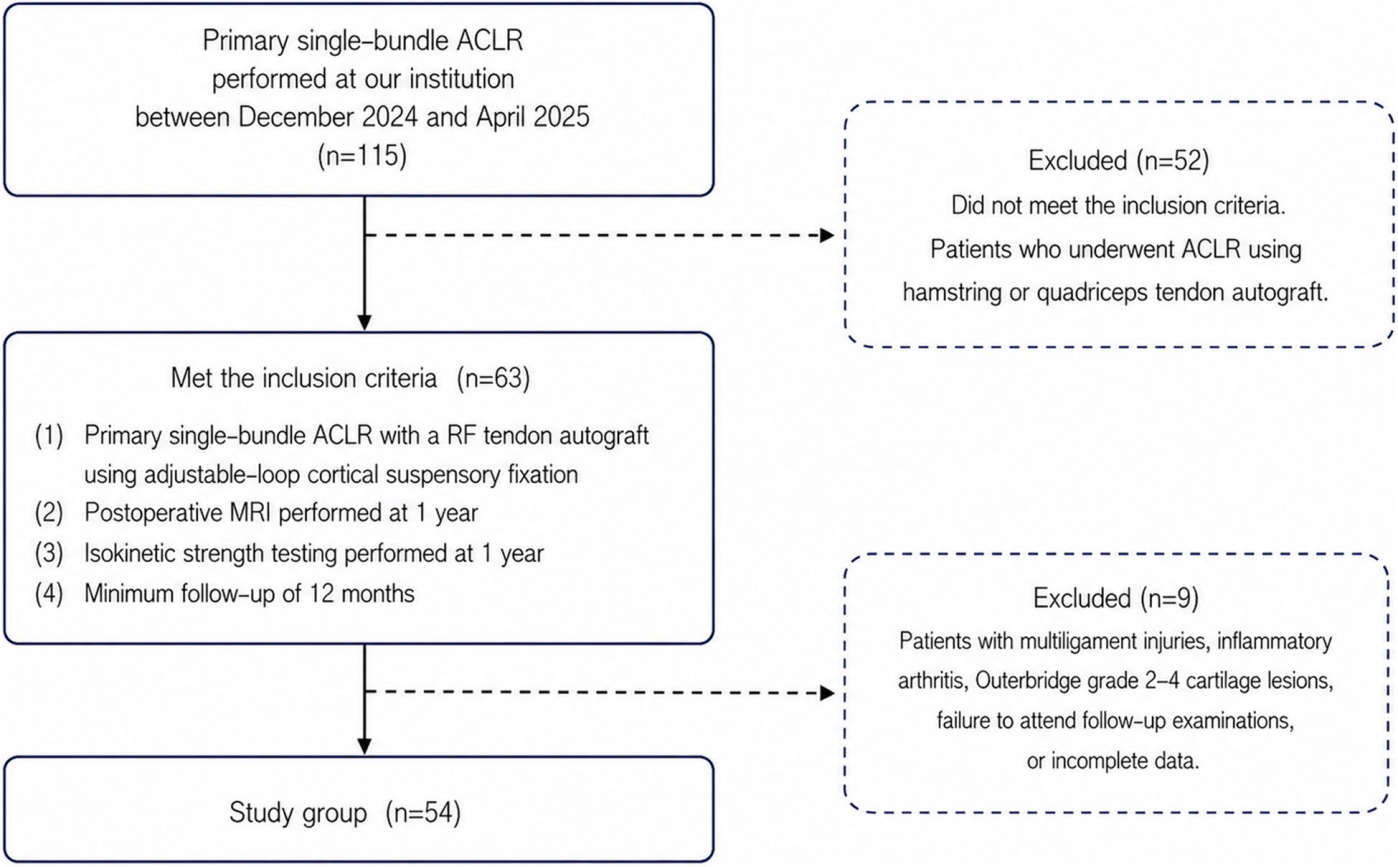
Flowchart of patients

This study was designed as a descriptive single-cohort evaluation to investigate postoperative donor-site morbidity associated with RF tendon harvest. After obtaining ethics committee approval for the study protocol, we retrospectively reviewed the records of 115 patients who underwent single-bundle ACLR at our institution between December 2024 and April 2025. The inclusion criteria were as follows: (1) primary single-bundle ACLR with a RF tendon autograft using adjustable-loop cortical suspensory fixation; (2) postoperative magnetic resonance imaging (MRI) performed at one year; (3) isokinetic strength testing performed at one year; and (4) a minimum follow-up of 12 months. Patients with multiligament injuries, inflammatory arthritis, Outerbridge grade 2–4 cartilage lesions, failure to attend follow-up examinations, or incomplete data were excluded. According to these criteria, 54 patients were included in the final analysis (Fig. 1).

### Surgical technique and rehabilitation

All patients underwent anatomic single-bundle ACLR using a RF tendon autograft with suspensory fixation. Associated meniscal and chondral lesions were addressed before reconstruction. The harvested RF tendon (24–30 cm) was quadrupled and fixed with an adjustable-loop cortical suspensory device on the tibial side and a fixed-loop cortical button on the femoral side.

The femoral tunnel was created anatomically through the anteromedial portal. After drilling a 4.5-mm guide tunnel, tunnel length was measured, and a socket matching the graft diameter was reamed, allowing a 6- to 8-mm space for button flipping. A complete outside-in tibial tunnel was then created at the centre of the native footprint. The graft was passed through the tibial tunnel into the femoral socket and tensioned at 20° of knee flexion. After 30 cycles of knee flexion–extension, graft tension was reassessed, re-tensioned on the tibial side, and secured with additional nonsliding knots.

All patients followed the same rehabilitation protocol, consisting of partial weightbearing with crutches for four weeks, gradual increase in knee flexion, and achievement of full range of motion by six weeks. Return to sports was permitted between nine and 12 months postoperatively.

### Radiological evaluation

Magnetic resonance imaging of both thighs was performed using a 1.5-T scanner (MAGNETOM Sola, Siemens Healthineers, Erlangen, Germany). For volumetric muscle assessment, axial three-dimensional proton density (PD) isotropic images were acquired using a SPACE sequence. Imaging parameters were as follows: repetition time (TR), 400 ms; echo time (TE), 17 ms; field of view (FOV), 400 mm; slice thickness, 1.0 mm; base resolution, 256; phase resolution, 100%; slice resolution, 100%; bandwidth, 476 Hz/pixel; echo spacing, 4.18 ms; turbo factor, 55; echo train duration, 234 ms; number of averages, 1; and concatenations, 1. Imaging was performed in the transverse plane with a 3D isotropic acquisition consisting of a single slab with 288 slices per slab. The acquired voxel size was 1.6 × 1.6 × 1.0 mm. Acquisition time was approximately seven to eight min, depending on the individual examination.

**Fig. 2:**
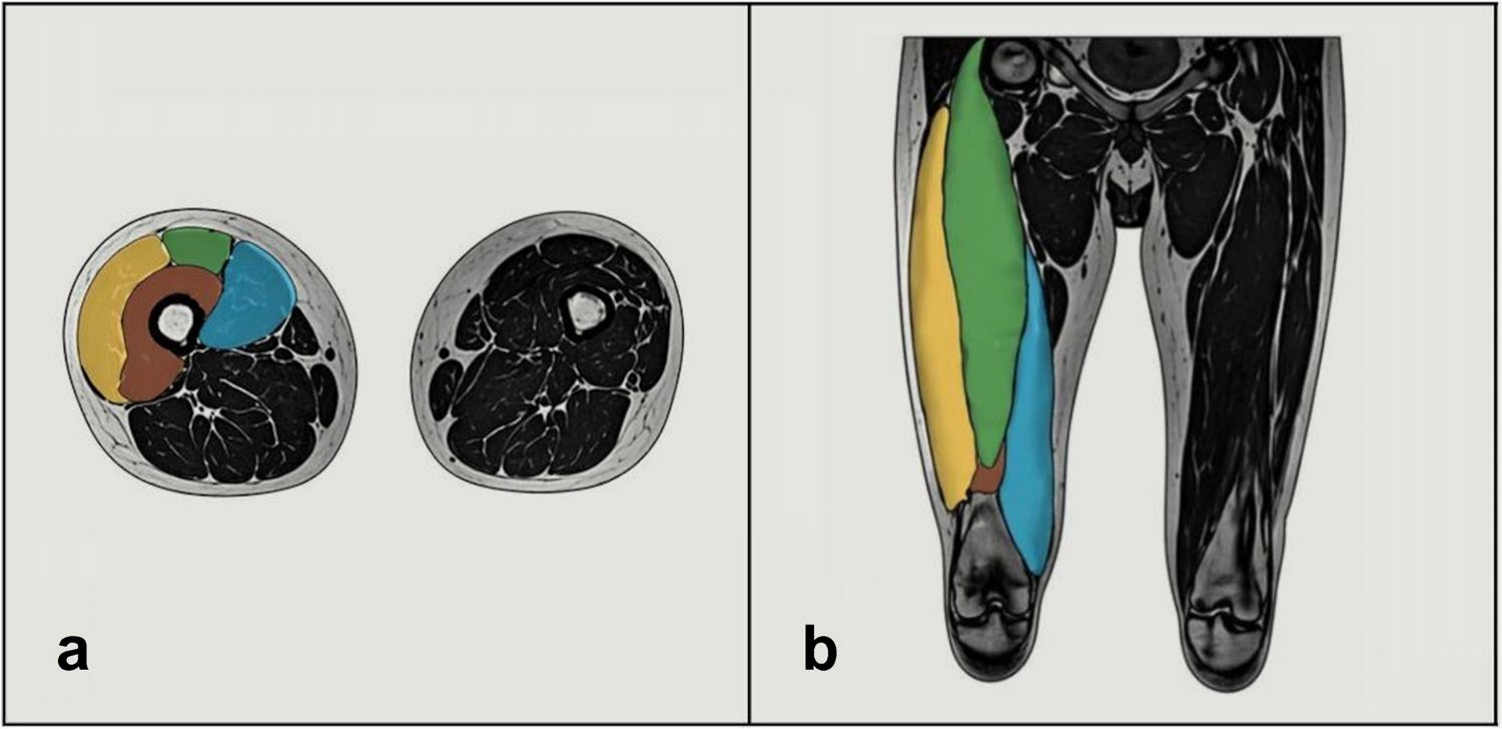
Volumetric measurement of quadriceps muscles on magnetic resonance imaging (MRI). Axial MRI slice demonstrating manual segmentation of the individual quadriceps muscles: rectus femoris, vastus lateralis, vastus intermedius, and vastus medialis **a**. Three-dimensional reconstruction of the segmented quadriceps muscles, enabling volumetric measurements **b**

For post-processing, MR images were transferred in DICOM format to the open-source medical image processing software 3D Slicer (version 5.10) for manual segmentation and volumetric analysis. Segmentation was performed using the Segment Editor module on axial PD isotropic images. In each participant, the quadriceps muscles of both thighs, including the RF, vastus lateralis (VL), vastus medialis (VM), and vastus intermedius (VI) were segmented separately. Each muscle was manually outlined slice by slice on consecutive images along its visible extent. Following completion of segmentation, three-dimensional models were reconstructed from the segmented datasets, and muscle volumes were calculated individually for each muscle. Separate volumetric measurements were obtained for the right and left thighs, and the analysis was based on individual muscle volumes (Fig. 2).

### Isokinetic, clinical and functional outcome measures

The following baseline characteristics were recorded: age, sex, operative side, body mass index (BMI), interval from injury to surgery, and duration of follow-up. Patient-reported outcomes at one year postoperatively were assessed using the International Knee Documentation Committee (IKDC) subjective knee form and the Marx Activity Rating Scale. Knee stability was evaluated at the same follow-up using the pivot-shift test and side-to-side anterior laxity measured with a KT-1000 arthrometer (Medmetric).

Knee extension (Ex) and flexion (Flx) strengths were measured using an isokinetic dynamometer (Humac Norm, CSMI, Stoughton, MA, USA) in con/con contraction mode at angular velocities of 60°/s (5 repetitions) and 240°/s (15 repetitions). The protocol included 4 practice trials (15-s rest) prior to each test velocity and a 30-s rest interval between velocities. Verbal encouragement was provided during the tests, and peak torque values (Nm) were recorded.

Limb function was evaluated using the single-leg hop test (SLHT) battery—single hop (SH), triple hop (TH), and crossover hop (CH)—performed along a standardized 6-m line. Participants initiated each test on the testing limb and achieved a controlled, balanced landing. The SH consisted of one maximal hop, whereas the TH and CH involved three consecutive hops, with the CH performed while crossing a reference line. Two trials were conducted for each test with a 30-s rest interval, and the maximum distance was recorded. Trials were repeated in case of rule violations (e.g., loss of balance or contralateral limb touchdown).

To minimize selection bias, consecutive patients meeting the predefined inclusion criteria were included. Measurement bias was reduced by using standardized protocols for MRI acquisition, isokinetic testing, and functional assessments. In addition, all MRI segmentations were performed using a consistent methodology, and both intraobserver and interobserver reliability were evaluated.

## Statistical analysis

All statistical analyses were performed using SPSS (version 22.0; IBM Corp). Continuous variables were expressed as mean ± standard deviation. Normality of data distribution was assessed using the Shapiro–Wilk test. Comparisons between the operative and contralateral sides were performed using paired-samples t tests. Effect sizes (Cohen d) with 95% confidence intervals were calculated to determine the magnitude of differences. One-way analysis of variance (ANOVA) was used to compare limb symmetry indexes (LSI) and quadriceps muscle volume asymmetry. An intraclass correlation coefficient of > 0.8 was considered excellent, and a value between 0.5 and 0.8 was considered good. Power analysis demonstrated that a sample size of 54 patients was sufficient to detect a moderate effect size (Cohen d = 0.60) with a type I error rate of 5% and 80% statistical power. Statistical significance was set at *p* < 0.05.

## Results

### Patient characteristics

Overall, 54 patients (34 male, 20 female) who underwent ACLR using a quadrupled RF tendon autograft with adjustable suspensory fixation were included in the study. The mean age at surgery was 28.1 ± 4.3 years, and the mean body mass index was 24.7 ± 2.6 kg/m^2^. The mean interval from injury to surgery was 3.8 ± 1.7 months, and the mean follow-up duration was 15.7 ± 4.2 months. Detailed demographic and operative data are presented in Table 1.

**Table 1:** Patient demographics and operative characteristics

Age, y	28.1 ± 4.3
Sex, M/F	34 (63.0%)/20 (37.0%)
Side, R/L	29 (53.7%)/25 (46.3%)
BMI, kg/m2	24.7 ± 2.6
Time from injury to surgery, m	3.8 ± 1.7
Graft Size	9.2 ± 1.3
Medial meniscus	
Normal, *n*(%)	25 (46.3%)
Repaired, *n*(%)	19 (35.2%)
Partial meniscectomy, *n*(%)	10 (18.5%)
Lateral meniscus	
Normal, *n*(%)	27 (50.0%)
Repaired, *n*(%)	21 (38.9%)
Partial meniscectomy, *n*(%)	6 (11.1%)
Follow-up time, m	15.7 ± 4.2

*M*: male, *F*: female, *R*: right, *L*: left, *BMI*: body mass index Data are presented as mean ± SD or *n*(%)

At final follow-up, the mean IKDC subjective score was 84.1 ± 9.7, and the mean Marx Activity Scale score was 7.1 ± 3.2. The mean side-to-side anterior tibial translation difference was 1.8 ± 1.2 mm. Median pivot-shift grade was 1 (interquartile range, 1–2). Clinical outcomes are summarized in Table 2.

**Table 2:** Clinical outcomes at final follow-up

IKDC score	84.1 ± 9.7
Marx Activity Scale	7.1 ± 3.2
ATTD, mm	1.8 ± 1.2
Pivot shift grade	1 (1–2)

Data are presented as mean ± standard deviation, except for pivot-shift grade, which is presented as median (interquartile range). ATTD: anterior tibial translation side-to-side difference, IKDC: International Knee Documentation Committee Subjective Knee Form

Reliability analysis demonstrated good to excellent agreement for all radiological measurements. Intra-observer intraclass correlation coefficients ranged from 0.82 to 0.87, while inter-observer values ranged from 0.79 to 0.83 (Table 3).

**Table 3:** Intra-class correlation coefficient for intra-observer and inter-observer reliability

	Intra-observer (95% CI)	Inter-observer (95% CI)
Rectus Femoris Volume	0.84 (0.71–0.93)	0.81 (0.68–0.91)
Vastus Lateralis Volume	0.87 (0.76–0.94)	0.83 (0.70–0.92)
Vastus Intermedius Volume	0.82 (0.69–0.91)	0.79 (0.65–0.89)
Vastus Medialis Volume	0.86 (0.74–0.94)	0.80 (0.67–0.90)

### Radiological results

**Fig. 3:**
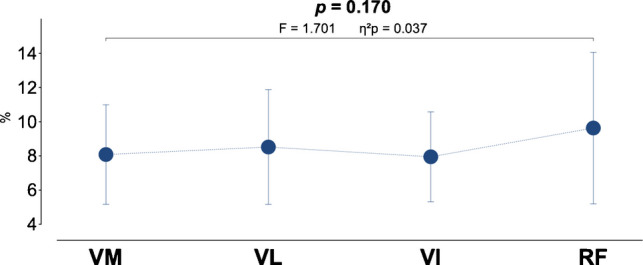
Intermuscular differences in quadriceps muscle volume at 1-year follow-up. Percentage differences between operated and contralateral limbs are shown for rectus femoris (RF), vastus lateralis (VL), vastus intermedius (VI), and vastus medialis (VM), with no significant between-muscle difference (F = 1.701, *p* = 0.170, η.^2^*p* = 0.037)

At one year follow-up, all quadriceps muscle volumes were significantly lower in the operated limb than in the contralateral limb (*p* < 0.001 for all). The mean side-to-side deficits were 9.63% for the RF, 8.52% for the VL, 7.95% for the VI, and 8.08% for the VM. No significant difference was found among the percentage deficits of the four muscles (F = 1.701, *p* = 0.170, η^2^*p* = 0.037), indicating a relatively uniform pattern of quadriceps atrophy (Fig. 3). Detailed radiological findings are presented in Table 4.

**Table 4:** Quadriceps muscle volumes and percentage differences at 1-year follow-up

	Operated limb volume (cm^3^)	Contralateral limb volume (cm^3^)	% Difference	t	*p*		95% CI	
Muscle						ES	LB	UB
Rectus Femoris	193.1 ± 27.22	213.8 ± 28.70	9.63	−11.42	<.001	−1.95	−2.53	−1.37
Vastus Lateralis	554.7 ± 67.20	605.9 ± 65.14	8.52	−14.41	<.001	−2.47	−3.14	−1.78
Vastus Intermedius	450.0 ± 45.66	488.8 ± 46.61	7.95	−16.70	<.001	−2.86	−3.62	−2.09
Vastus Medialis	380.2 ± 45.98	413.4 ± 46.41	8.08	−16.36	<.001	−2.80	−3.55	−2.04

Data are presented as mean ± standard deviation (SD). t: paired-samples t test result; ES: effect size; LB: lower bound; UB: upper bound. Differences in asymmetry among the quadriceps muscles were additionally analyzed, demonstrating no significant difference (F = 1.701, *p* = 0.170, η^2^*p* = 0.037)

### Isokinetic, clinical and functional results

In the comparison of isokinetic strength and hop performance, no significant differences were found between the operative and contralateral sides in 60°/s extension, 60°/s flexion, and 240°/s flexion strengths, as well as in the SH and CH tests (*p* > 0.05). However, significant differences were observed in favour of the contralateral limb for 240°/s isokinetic extension strength (*p* = 0.03, ES = 0.37) and TH performance (*p* < 0.001, ES = 0.65). Differences in limb symmetry indexes (LSI) were additionally analyzed with One-way ANOVA, demonstrating no significant difference (F = 1.960, p = 0.48) (Table 5). This confirms the consistency of the findings, indicating that targeted symmetry levels adequate for return to sport (RTS) were achieved across all evaluated strength and functional parameters.

**Table 5:** Isokinetic strength and functional performance test results

	Operative Side	Contralateral Side	t	p	LSI (%)	ES	95% CI	
							LB	UB
60°/sec Ex (Nm)	181.12 ± 45.15	170.74 ± 45.71	1.49	0.14	96	0.25	−0.08	0.59
240°/sec Ex (Nm)	94.12 ± 26.36	86.76 ± 24.74	2.18	0.03	94	0.37	0.02	0.72
60°/sec Flex (Nm)	109.91 ± 24.98	103.53 ± 21.72	2.41	0.07	95	0.41	0.16	0.76
240°/sec Flex (Nm)	64.62 ± 19.65	62.32 ± 13.73	2.18	0.31	100	0.37	0.12	0.72
SL (cm)	118.03 ± 15.75	120.29 ± 18.61	−0.63	0.53	100	0.11	−0.44	0.23
TH (cm)	378.50 ± 59.90	422.09 ± 69.07	−3.84	<.001	91	0.65	−1.02	−0.28
CHD (cm)	318.94 ± 58.30	339.41 ± 77.66	−1.65	0.11	97	−0.28	−0.62	0.06

Data are presented as mean ± standard deviation (SD). t: paired-samples t test result; ES: effect size; LB: lower bound; UB: upper bound; Ex: extension; Flex: flexion; Nm: Newton-meter; SL: single-leg hop for distance; TH: triple hop for distance; CHD: crossover hop for distance

## Discussion

The most important finding of the present study was that ACL reconstruction using a RF tendon autograft provided favorable clinical and functional outcomes at one year postoperatively. Patients demonstrated restoration of limb symmetry with satisfactory isokinetic strength recovery. Although overall quadriceps muscle volumes were reduced postoperatively, RF graft harvest did not result in selective RF muscle atrophy, nor was it associated with clinically relevant strength deficits at one year.

Clinical evidence regarding the use of RF tendon grafts in primary ACL reconstruction remains limited. Rego et al. [13] reported excellent short-term outcomes in a case series of 80 primary ACL reconstructions, with a mean Lysholm score of 97.1, a low complication rate, and no graft failures at final follow-up, supporting the safety and effectiveness of the RF graft option. In the revision setting, Huber et al. [14] reported that RF tendon and hamstring tendon autografts achieved similar patient-reported outcomes, suggesting that the RF tendon is also a reliable graft source for revision ACL reconstruction. Consistent with these findings, our study demonstrated favourable one year outcomes after primary ACL reconstruction using a RF autograft, with a mean IKDC score of 84.1, Marx Activity Scale score of 7.1, side-to-side laxity difference of 1.8 mm, and a median pivot-shift grade of 1. Collectively, these results further support the RF tendon as a safe and effective graft option for ACL reconstruction.

The measurement of muscle volume remains methodologically challenging, and the interpretation of side-to-side differences is not straightforward [15–17]. Although magnetic resonance imaging is considered the reference standard, it is labour-intensive and subject to segmentation-related variability [16]. Importantly, defining physiological asymmetry is essential before classifying observed differences as pathological. Kulas et al. [15] reported that mean quadriceps asymmetries in healthy individuals ranged from − 3.0% to 6.0%, with 95% limits of agreement reaching approximately ± 8.8% at the whole muscle level and even greater variability at the individual muscle level, with a substantial proportion exceeding 10% asymmetry. In addition, accumulating evidence suggests that the contralateral limb may not represent a truly normal reference after ACL injury and reconstruction because of bilateral neuromuscular adaptations involving sensorimotor control, corticospinal tract structure, and brain activation [18–21]. Konishi et al. [22] demonstrated reduced quadriceps muscle volumes in both injured and uninjured limbs compared with healthy controls, indicating that side-to-side comparisons may underestimate the true extent of postoperative atrophy. In our study, persistent volume deficits were observed across all quadriceps components at one year postoperatively. Given the potential for bilateral neuromuscular alterations after ACL reconstruction, these consistent deficits likely represent clinically meaningful postoperative quadriceps muscle volume loss.

Quadriceps atrophy after ACL injury and reconstruction has been well documented, although the magnitude and clinical relevance of muscle loss remain variable [22, 23]. In a systematic review, Birchmeier et al. [23] found that most studies reported smaller quadriceps cross-sectional area or muscle volume in the reconstructed limb compared with the contralateral side, but only 36% demonstrated clinically meaningful differences. Similarly, Konishi et al. [22] reported significantly reduced quadriceps femoris muscle volume and lower torque values on the injured side compared with the uninjured side. Our results also confirm the presence of sustained quadriceps volume deficits at one year follow-up across all quadriceps muscles. However, despite graft harvest from the RF, no disproportionate loss was observed in the RF muscle compared with the VM, VL, and VI, suggesting that tendon harvest did not produce selective donor-site atrophy. This finding may be explained by the unique anatomy of the RF tendon [24]. During graft harvest, only the one-third posterior fascia of the RF is typically harvested, while the central aponeurosis and the proximal direct and indirect tendinous attachments are preserved [9]. Furthermore, the RF has a complex internal architecture with separate superficial and deep tendinous components, which may help maintain force transmission and muscle integrity after partial tendon harvest [24, 25]. These anatomical characteristics may explain the absence of clinically relevant selective RF muscle volume loss following graft harvest.

During the first year following surgery, recovering quadriceps strength and limb symmetry are among the primary objectives, as LSI values of 90% or higher are widely accepted as a key criterion for return-to-sports [26]. However, the literature presents conflicting results regarding the success of achieving these targeted symmetry values after ACLR[27–29]. While Shi et al. [29] reported persistent extension deficits and plateaued LSI values six to 12 months post-surgery; Legnani et al. [28] indicated in their study that most patients met the 90% LSI criterion by the 12th postoperative month. These conflicts in the literature may be directly influenced by variables such as the graft type used, the patients' athletic background, concomitant pathologies associated with the ACL injury, and variations in additional clinical findings during the postoperative period [30]. The findings obtained from our study demonstrated that LSI values exceeded the 90% threshold in all isokinetic and hop tests at the 12-month evaluation, with no statistically significant difference between extremities in most strength and functional measures. However, residual deficits persisted in high-velocity extension strength and triple-hop performance. These findings may reflect persistent impairments in explosive quadriceps function, neuromuscular control, and rapid force generation, which are more sensitively detected during high-speed functional tasks and may persist despite satisfactory recovery in routine strength parameters. Therefore, these residual deficits may be related to the demanding nature of high-level dynamic activities rather than isolated donor-site morbidity associated with RF tendon harvest.

## Limitations

This study has several limitations. First, its retrospective design may have introduced selection bias and limited control over potential confounding variables. Second, although the MRI-based muscle volume measurements demonstrated high intraobserver and interobserver reliability, the accuracy of volumetric assessment may still depend on the segmentation technique used [17]. Third, the clinical follow-up period was relatively short and limited to the first postoperative year. Therefore, potential changes in donor-site morbidity, muscle recovery, or functional performance beyond this period remain unknown. Fourth, the absence of a comparative graft cohort limits direct comparisons with other established autograft options. Fifth, the use of the contralateral limb as a reference control has inherent limitations, as bilateral neuromuscular adaptations and generalized deconditioning after ACL reconstruction may influence side-to-side comparisons [18, 20]. Therefore, limb symmetry indexes may overestimate postoperative recovery due to impaired contralateral limb function. Finally, the present analyses were focused on outcomes at a single postoperative time point during the rehabilitation process and therefore cannot fully reflect the long-term postoperative changes.

## Conclusion

Primary ACLR using a quadrupled RF tendon autograft resulted in favourable one year clinical and functional outcomes with restoration of limb symmetry. Quantitative MRI analyses demonstrated that RF graft harvest did not result in selective muscle volume loss or clinically relevant strength deficit at one year postoperatively.

## Data Availability

No datasets were generated or analysed during the current study.
